# Some Points about Head Injuries

**Published:** 1909-01-23

**Authors:** 


### Surgery.

### SOME POINTS ABOUT HEAD INJURIES.

### I.—CONCUSSION.

*Nullum vulnus capitis contemnendum est.* This is an old saying and a very true one. Any patient who has received a blow on the head of sufficient violence to render him even temporarily unconscious must be regarded as having suffered a grave surgical injury, and if such patients are kept in bed under careful observation many a serious error will be avoided.

In these cases the brain is in a state of shock. We are not concerned here with the relative merits of the hypotheses to account for the serious nature of the clinical condition, in view of the fact that no gross lesion of the brain can be found in the post-mortem room. The old explanation was that it was due to molecular disturbance of the brain, the so-called *commotio cerebri*. But in more recent times it has been suggested that it is either due to multiple cerebral hæmorrhages which are so minute as to escape observation, or that the injury causes a cone of depression on the brain. The vault of the skull possesses a certain degree of elasticity; this has a double effect—the brain at the point of injury is driven inwards, causing a local anæmia, and cerebro-spinal fluid is driven out, the normal pressure on the medullary centres being thereby modified. The exact nature of the state of affairs is likely, however, to remain a matter for conjecture since few uncomplicated cases of concussion end in the post-mortem room, and when they do so nothing definite can be found to account for the symptoms. When one is called to a case of concussion, one usually reaches the patient some little time after the infliction of the injury. In order to find out the nature of the accident, reliance must necessarily be placed on the evidence of any eye-witnesses who happened to be present, since the patient, even if he has by that time recovered consciousness, cannot give any details. All that one usually extracts is that the patient either fell or was struck upon his head. That, however, is sufficient.

As has been said, the patient may by this time have recovered consciousness, or he may still be completely insensible. In the former case he will probably have vomited, since vomiting is often the first sign of returning consciousness. He may then be dazed or even comparatively sensible, but is in nearly all cases completely unable to recall any detail of the actual accident.

It would seem, therefore, that the diagnosis ought to be fairly obvious; but the difficulty is greatly increased in the hospital class of case because many of them are confirmed alcoholics, and the question arises as to whether their condition is the result of alcoholic excess only, or injury, or both. If there be the slightest doubt, the only safe thing to do is to keep the patient under observation until the question is definitely settled. Mistakes have frequently occurred through lack of this precaution, and in one case in the writer's knowledge a patient was sent out of a hospital as being merely drunk, and was taken to the police-station. He died in the cells during the night, and at the autopsy it was discovered that he had a fracture of the base of the skull. Moreover, in an unconscious man the only evidence of alcoholic excess is obtained from the smell of his breath, and a patient whose habits are perfectly blameless in this respect may be suspected quite unjustly on this evidence alone. For the first thing that a layman does on seeing a patient who has had a severe injury is to pour brandy down his throat. By the lay world brandy is regarded as a panacea in all cases of emergency. It is, of course, nothing of the sort, and frequently, as in this case, does more harm than good, as will be explained later. It is the duty of every medical man to impress upon the public that it is impertinent meddling on their part to administer drugs to an injured individual on their own responsibility. But, it may be asked, what should a non-medical man do in the face of such an emergency. The answer is simple. He should leave the patient alone, and should concern himself only with finding suitable means for conveying him to the nearest doctor. In London the police arrangements are so admirable that there is almost always an ambulance at no great distance; but in the country some more impromptu means of conveyance must be found. A gate or a door taken off its hinges, or even a hurdle, answers the purpose admirably. The layman does not know what ought to be done, and if he did, the probability is that he has not the necessary skill nor training to do it. As it is, he rushes to the patient and administers brandy, because, he argues, it is better to give brandy than to do nothing at all.

Well, then, let us say that a patient has received a blow on the head sufficiently severe to render him unconscious, and has been carried someway to the doctor's house. He will be found to be suffering from surgical shock, that is to say the surface of the body will be cold and clammy and the temperature will be found to be subnormal. If, however, he happens to be seen immediately after the accident these phenomena will not have made their appearance.

He lies motionless on his back and his respirations are shallow and sighing. The pulse is weak and slow. The pupils, in an uncomplicated case of concussion, are equal. Generally they are constricted also, though they may occasionally be dilated. The greater frequency of myosis is due to the fact that the tonic power of the constrictors is greater than that of the dilators, when the voluntary control is removed. For the same reason the pupils are contracted during sleep. The reflexes, as demonstrated by the condition of the knee-jerk, are sluggish, and may be completely absent. Occasionally, but not often, there is incontinence, owing to relaxation of the sphincters. Consciousness usually returns in from six to twelve hours, and is, as has been said, often preceded by vomiting. If the interval is longer, it is presumptive evidence that there is also some contusion of the brain.

